# Twinning-induced strain hardening in dual-phase FeCoCrNiAl_0.5_ at room and cryogenic temperature

**DOI:** 10.1038/s41598-018-28784-1

**Published:** 2018-07-13

**Authors:** M. Bönisch, Y. Wu, H. Sehitoglu

**Affiliations:** Department of Mechanical Science and Engineering, University of Illinois at Urbana-Champaign, 1206W. Green St., Urbana, IL 61801 USA

## Abstract

A face-centered-cubic (fcc) oriented FeCoCrNiAl_0.5_ dual-phase high entropy alloy (HEA) was plastically strained in uniaxial compression at 77K and 293K and the underlying deformation mechanisms were studied. The undeformed microstructure consists of a body-centered-cubic (bcc)/B2 interdendritic network and precipitates embedded in 〈001〉-oriented fcc dendrites. In contrast to other dual-phase HEAs, at both deformation temperatures a steep rise in the stress-strain curves occurs above 23% total axial strain. As a result, the hardening rate associated saturates at the unusual high value of ~6 GPa. Analysis of the strain partitioning between fcc and bcc/B2 by digital image correlation shows that the fcc component carries the larger part of the plastic strain. Further, electron backscatter diffraction and transmission electron microscopy evidence ample fcc deformation twinning both at 77K and 293K, while slip activity only is found in the bcc/B2. These results may guide future advancements in the design of novel alloys with superior toughening characteristics.

## Introduction

### The promise of dual-phase high entropy alloys

The last two decades witnessed a surging interest in high entropy alloys (HEAs) by scientists and engineers making them one of the most widely studied alloy class nowadays. This excitement is largely rooted upon their outstanding mechanical attributes, above all high ductility at moderate to extremely low temperatures and remarkable toughness^1–6^.

Early studies aimed at preparation of single phase equiatomic HEAs exhibiting face-centered-cubic (fcc), body-centered-cubic (bcc) and hexagonal-close-packed (hcp) structures eliminating secondary (and tertiary) phases. However, the complexity of the phase formation in multi-component systems often prevented this goal or drastically limited the admissible composition range. And so, in recent years a paradigm shift has taken place that puts the focus on dual-phase (or multi-phase) HEAs and intentionally takes advantage of their heterophase nature to achieve superior mechanical attributes. Concurrently, the strong emphasis on equiatomic composition (which many early studies adopted) has been widely relaxed leading to a more integral exploration of the central zones of multi-dimensional composition space including the effects of minor alloying additions in the single digit percent range recently^2,7–13^.

**Figure 1:**
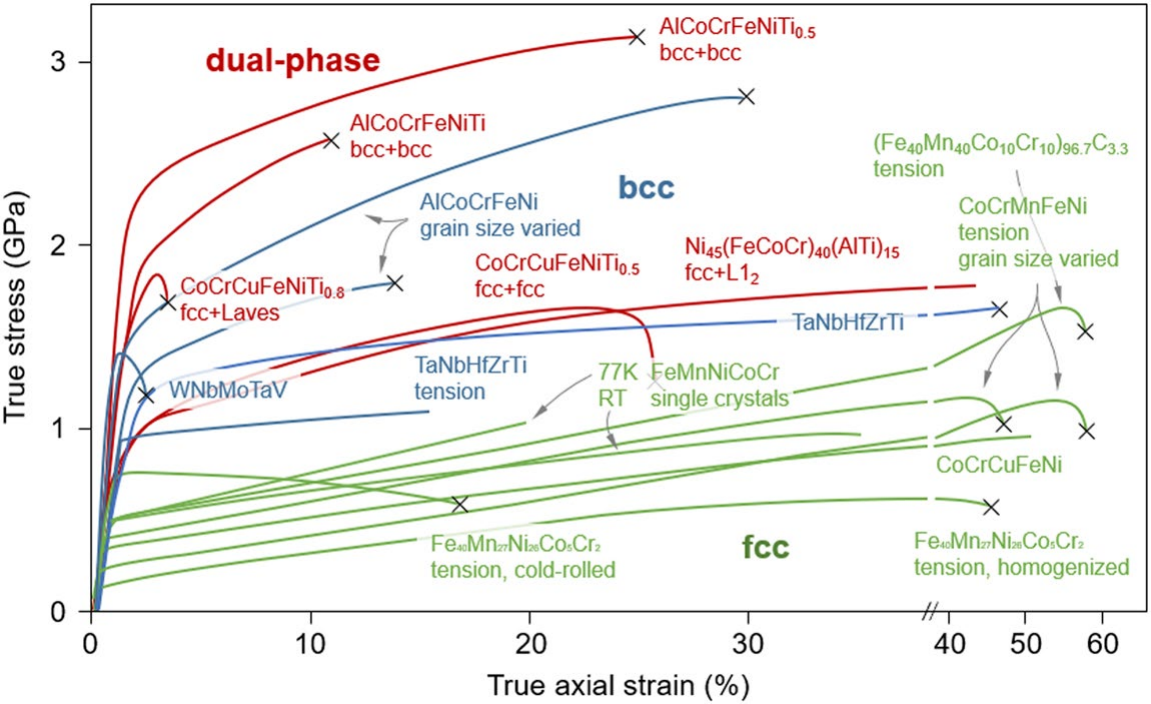
Comparison of stress-strain behavior of single phase fcc and bcc vs. dual-phase HEAs^2,6,7,9,13,14,30,38,47,48^. Curves shown are compressive unless otherwise stated. Crosses indicate failure by fracture.

The mechanical properties of HEAs are to a large part governed by their crystal structures, as illustrated in Fig. 1. The combination of Fe, Mn, Ni, Co and Cr in an equi-atomic fashion forms a pure fcc solid solution. This class of materials has been found to exhibit superior ductilities (in some cases up to 80% nominal strain) at cryogenic temperatures yet low strength (typically <800 MPa). The addition of sufficient amount of bcc stabilizers, e.g. Al, Ta, W, V and Mo, facilitates the formation of a single-phase bcc structure. These bcc alloys have increased strengths, e.g. 2.5 GPa in the case of AlCoCrFeNi^14^, relative to fcc alloys, though tend to be more brittle or show lower strains at fracture. Dual-phase HEAs in turn may provide a solution to overcome the strength-ductility trade-off. For example, the maximum stress level can reach 3 GPa and strain level ~25% in a AlCoCrFeNiTi_0.5_ alloys consisting of both bcc matrix and disordered bcc Widmanstätten phase^7^.

The quest for improved damage tolerance drives the development of new high-strength high-toughness materials, e.g. dual-phase HEAs, advanced (such as dual-phase, twinning-induced plasticity - TWIP, transformation induced plasticity - TRIP) steels^15–18^, Ti- and Cu-alloys^19–21^. The growing interest in dual-phase HEAs and in FeCoCrNiAl_x_ alloys in particular has valid reasons. For instance, the FeCoCrNiAl alloy can be regarded as a lightweight dual-phase high entropy superalloy with potential for high-temperature structural applications, e.g. aerospace engine components^12^. Owing to its high Fe and Al contents, FeCoCrNiAl is lighter and cheaper to fabricate than conventional super-alloys. Other varieties containing Ti in addition demonstrated excellent room temperature compressive strengths that were superior even to many bulk metallic glasses^7^. To date FeCoCrNiAl_x_ alloys have been studied mostly in terms of their metallurgy and basic mechanical quantities^8,12,14,22–25^.

Their microstructure and constitution of phases are very sensitive to the Al content and can be purposely varied from single-phase fcc (for x ≅ 0–0.4), to dual-phase fcc + bcc/B2 (for x ≅ 0.4–0.9) to single-phase bcc/B2 (for x ≅ 0.9–2.0)^8,22,23^.

The material studied here is a FeCoCrNiAl_0.5_ alloy, characterized by a two-phase microstructure consisting of 〈001〉-oriented fcc dendrites with fine bcc/B2 precipitates embedded and bounded by a bcc/B2 interdendritic. The intimate two-phase fcc + bcc/B2 microstructure is expected to entail complex heterogeneous strain partitioning between these phases because of their mechanical contrast^26^. Also, heterogeneous grain and phase distributions in addition to segregations (chemical gradients) resulting from the dendritic solidification during casting add to the intricacy of its stress-strain response. Our experiments reveal that this composition shows exceptional work hardening both at 293K and 77K, considerably higher than previously reported hardening rates for e.g. Fe-Mn and Hadfield steels^15,27^.

Based on the experimental observations elaborated below we attribute the substantial hardening to the combined action of these 3 contributions: (i) the fcc – bcc/B2 interphase boundaries resulting from the dendritic-interdendritic microstructure, (ii) extensive deformation twinning in fcc, (iii) fine and dense bcc/B2 precipitates inside the fcc dendrites.

In this paper, we focus on the central role that deformation twinning plays for the mechanical response of this alloy. In particular the onset of twinning in the fcc phase at high plastic strain produces a remarkable increase in the work hardening both at 293K and 77K. This results in an upward curvature in the stress-strain response of this class of alloys. In addition to measurements of the local strain by digital image correlation (DIC) and quantification of the plastic strain partitioning between fcc and bcc/B2 phases, electron microscopy illustrates extensive fcc twinning.

## Materials and Methods

Using the Bridgman technique an ingot of nominal composition FeCoCrNiAl_0.5_ (mole ratio) was prepared in Ar atmosphere. The effective composition was within 0.5 at.% of the nominal based on wet chemical analysis. After homogenization at 1373K for 24 h and annealing at 1123K for 1 h (both terminated by water quench) the crystallographic orientation of the fcc and bcc/B2 components was measured via electron backscatter diffraction (EBSD). While the fcc matrix exhibited a single orientation (corresponding to a single contiguous grain) with 〈001〉 aligned with the loading direction, the bcc/B2 components (interdendritic and precipitates) were clustered close to 〈001〉 and 〈011〉. Variations in the chemical composition between fcc and bcc/B2 components were measured via energy dispersive X-ray spectroscopy (Table 1). The dendritic fcc matrix is Ni and Al rich while the bcc/B2 interdendritic is Ni-rich and depleted of Co. The bcc/B2 precipitates on the other hand contain equal amounts of Fe, Co and Cr and are Al-poor.

**Table 1:** Elemental composition (in at.%) and volume fraction (in %) for each phase after 1 h at 850 °C and WQ.

Phase	Fe	Co	Cr	Ni	Al	vol.%
fcc (dendritic)	14.5 ± 0.3	18.6 ± 0.4	12.6 ± 0.2	33.7 ± 0.4	20.6 ± 0.3	87
bcc/B2 (interdendritic)	16.9 ± 0.3	9.7 ± 0.3	15.4 ± 0.2	31.0 ± 0.4	17.0 ± 0.4	13
bcc/B2 (precipitates)	24.5 ± 0.3	24.1 ± 0.4	24.4 ± 0.2	22.0 ± 0.4	5.0 ± 0.4	

The compression specimens were sectioned using electro-discharge machining (EDM) to a geometry of 4 mm × 4 mm × 8 mm. Surface damage from the cutting process and oxidation from the heat treatment were removed by grinding with abrasive paper up to 4000 grit. For EBSD analysis, the samples were vibration-polished. A speckle pattern was applied on the mirror-finished surface for *ex-situ* DIC strain measurements. Subsequently, compression tests were carried out at room temperature (293K) and in liquid nitrogen (77K). For deformation at 77K sample and grips were fully submerged in liquid nitrogen. The total axial strain for stress-strain curves was recorded with an extensometer and the local distribution of strain was measured through *ex-situ* high-resolution digital image correlation (DIC)^28^. For DIC, deformation was carried out incrementally. Images of the pristine and the strained states were recorded with an optical microscope having a resolution of 0.08 μm/pixel. Reference and deformed images were correlated using VIC-2D (Correlated Solutions, Inc.). To enlarge the area of observation, 25 images (5 × 5) were captured, which results in a region of 0.4 mm × 0.55 mm. Correlations were performed on each pair of reference and deformed images. The resultant DIC strain contours were stitched together using the procedure outlined by Carroll *et al*.^29^.

Specimens for transmission electron microscopy (TEM) were extracted from the pristine and deformed FeCoCrNiAl_0.5_ via focused ion beam (FEI Helios 600i DualBeam). Bright-field imaging, dark-field imaging and diffraction was carried out in a JEOL 2010 LaB_6_ and high-resolution imaging in a JEOL 2010F TEM.

### Data availability

All data generated or analyzed during this study are included in this published article.

## Experimental Results

In this section we present the key experimental observations documenting the stress-strain response of dual-phase FeCoCrNiAl_0.5_ in relation to its pristine vs. deformed microstructure. After characterizing the undeformed microstructure by EBSD and TEM, the influence of temperature (293K vs. 77K) on the strain-hardening rate is studied and the strain partitioning between fcc and bcc/B2 determined from DIC strain maps. EBSD and (high-resolution) TEM of deformed FeCoCrNiAl_0.5_ reveal deformation twinning in the fcc phase.

### Undeformed state

**Figure 2:**
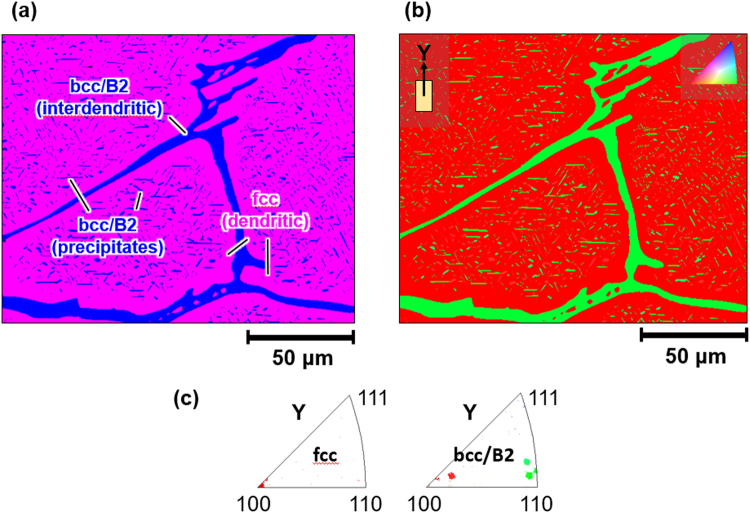
(**a**) Phase map and (**b**) orientation map of the undeformed microstructure after 1 h at 850 °C and WQ obtained via EBSD. (**c**) Inverse pole figures showing the orientation of the fcc (dendritic) and the bcc/B2 (interdendritic and precipitates) components.

**Figure 3:**
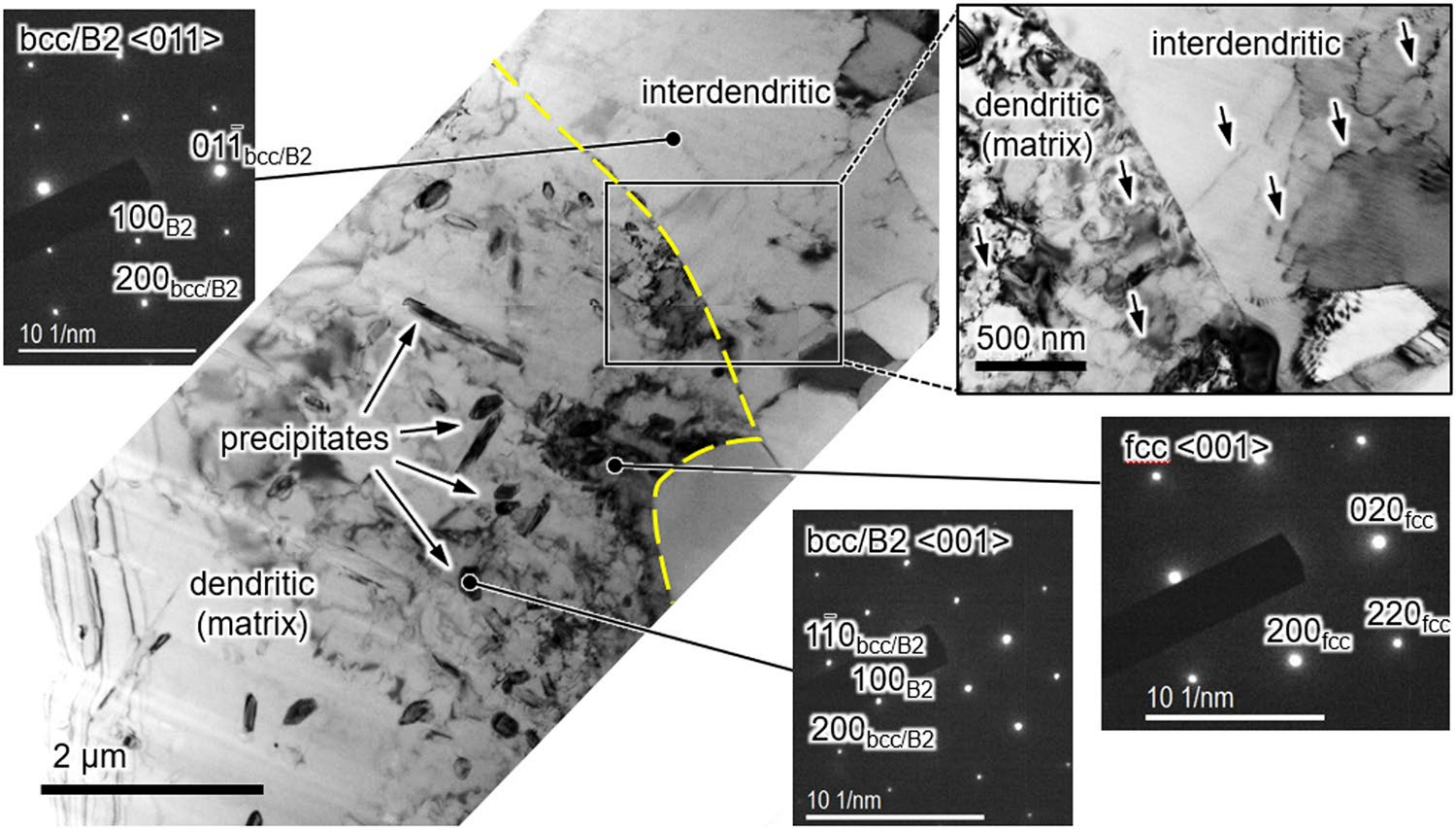
TEM bright-field images of the undeformed state (after 1 h at 850 °C and WQ) and SAED patterns of the dendritic matrix (fcc), interdendritic and precipitates (bcc/B2). The interdentritic-dendritic phase boundary is indicated by yellow dashes. Arrows in the magnified section point at dislocations.

Various studies have shown that the constitution of phases and their morphologies in FeCoCrNiAl_x_ alloys strongly depend on the Al content and thermal treatment^8,12,14,23,25,26^. The FeCoCrNiAl_0.5_ alloy under study here is a representative case for a dual-phase fcc + bcc/B2 microstructure, as demonstrated in Figs 2 and 3.

The EBSD phase map in Fig. 2a shows that the fcc matrix, occupying 87 vol.% (Table 1), forms dendrites, 200–500 μm in diameter, intersected by an open network of bcc/B2 interdendritic. Additionally, embedded in the fcc are fine platy bcc/B2 precipitates, their longer axis ranging from a few 100 nm to 15 μm and having a shorter axis (thickness) below ~2 μm, as indicated by EBSD and TEM. Through selected area electron diffraction (SAED) (Fig. 3) the crystal structure of the dendrites was identified with fcc and that of the precipitates and the interdendritic with bcc/B2. Superstructure reflections present in SAED patterns recorded from precipitates and interdendritic evidence at least partial chemical B2 order, in agreement with X-ray diffraction results on related alloy varieties^8,23^. TEM revealed that fcc dendrites and bcc/B2 interdendritic, too, contain grown-in dislocations and the interdendritic is further fragmented by small angle grain boundaries (Fig. 3).

Via EBSD the orientation of the fcc dendrites was determined as 〈001〉 along the compressive loading axis Y. The bcc/B2 (precipitates and interdendritic) exhibit varying orientations. (Fig. 2b,c). It is important to note that the fcc dendrites form a contiguous volume of constant crystallographic orientation as evidenced by EBSD. The bcc/B2 in contrast exhibits varying alignments relative to the compressive loading axis. Conceptually, these oriented matrix dual-phase crystals lie between single crystals (precipitate and secondary phase free) exhibiting a single orientation and dual-phase polycrystals, in which both phases exhibit spread-out orientation distributions with a large fraction of high angle grain boundaries. By choosing oriented matrix dual-phase crystals the impact of the secondary phase on the matrix and overall deformation response may be revealed in a controlled manner.

### Stress-strain responses

**Figure 4:**
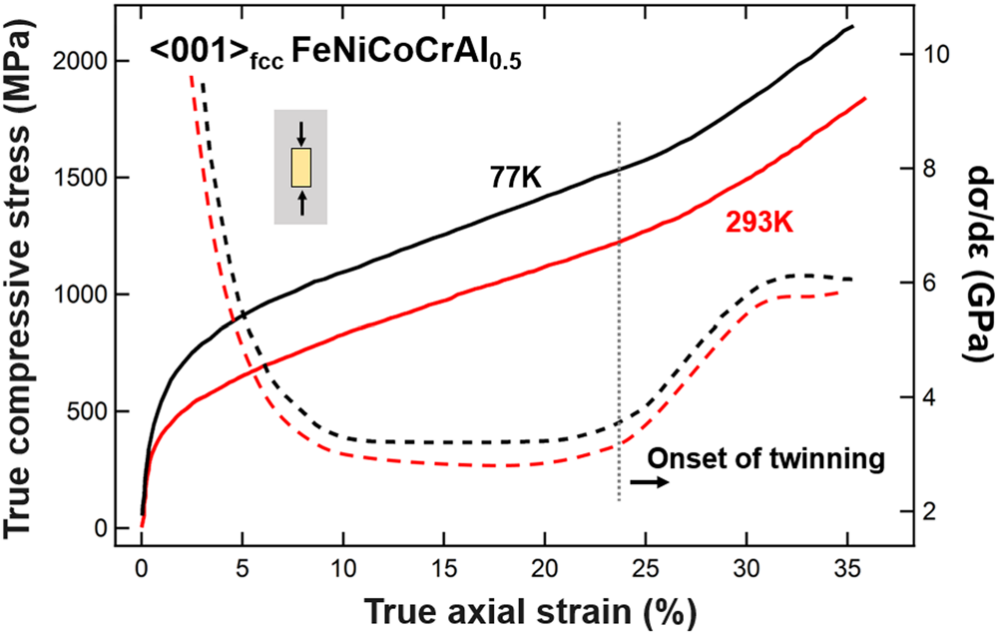
Compressive deformation behavior of 〈001〉_fcc_-oriented FeCoCrNiAl_0.5_. Solid and dashed lines show stress and hardening rate, respectively.

In light of previous studies evidencing deformation twinning in FeCoCrNiAl_x_ and FeCoCrNiMn at cryogenic temperature (77K)^3,4,24,30,31^ the macroscopic compressive stress-strain response of 〈001〉_fcc_-oriented FeCoCrNiAl_0.5_ was recorded both at 293K and 77K (Fig. 4). At both temperatures strong hardening is revealed from the outset of plastic deformation attaining 3–3.5 GPa between 10–23% axial strain. Most notably, beyond 23% axial strain the hardening rate increases by more than 2 GPa to reach ~6 GPa above 32% axial strain. Increasing the temperature from 77K to 293K leaves the overall behavior largely unaltered. It merely raises the stress level by 250–350 MPa, the variation of the hardening rate with strain remaining unaffected. In particular, the hardening rate increase of >2 GPa beyond 23% is present even at room temperature. As a result, the stress levels exceed 1800 MPa at a total axial strain of ~35% without failure at either temperature. We surmise that the reason for the high degree of strain hardening beyond 23% axial strain is deformation-induced twinning. This rise in the stress-strain slope is surprising and is to some extent reminiscent of the twinning-induced or martensitic transformation-induced upward stress-strain curvature in TRIP/TWIP steels, Ti- and Cu-alloys^15,16,18,20,21,32–34^. As will be shown later in this paper, deformation twinning indeed takes place in the fcc dendrites, providing a highly effective way for strain-hardening compared to slip-based mechanisms^30^. It is furthermore important to mention that the 〈001〉_fcc_-oriented FeCoCrNiAl_0.5_ exhibits promising ductility in tension. Tensile specimens deformed at 293K yielded at ~420 MPa and fractured at ~740 MPa and ~10% total tensile axial strain.

**Figure 5:**
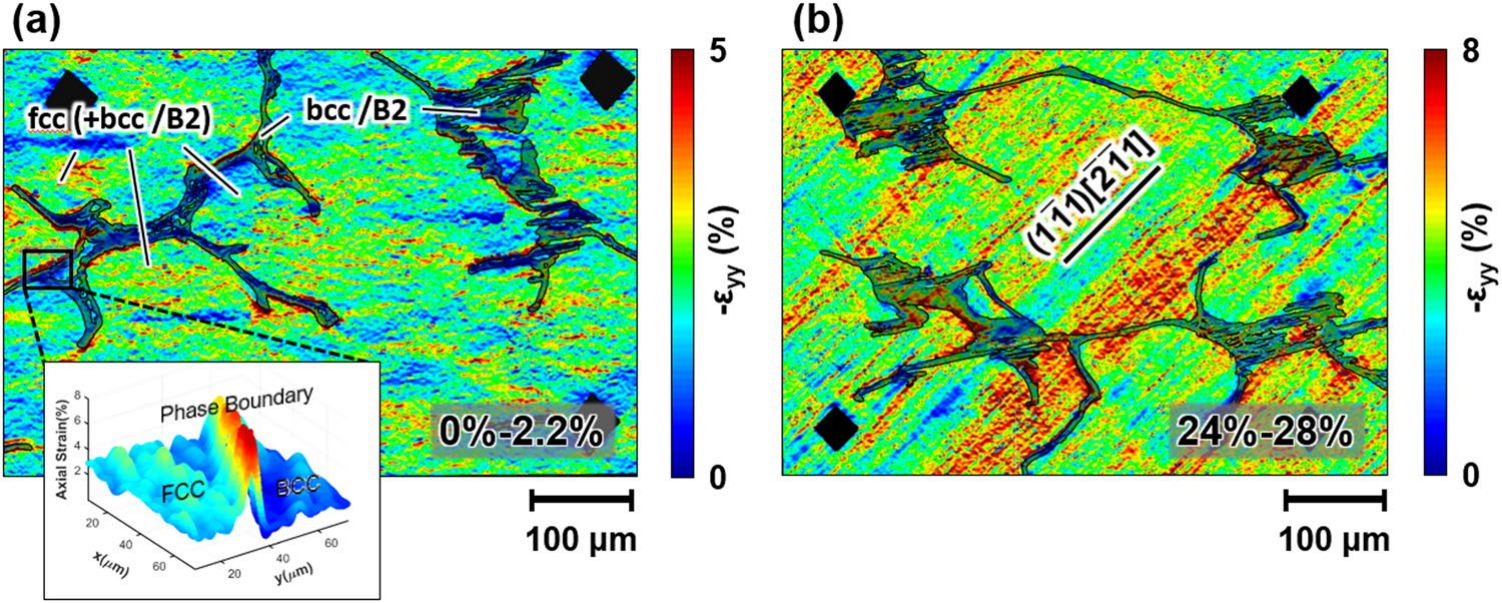
DIC strain maps showing the increase in axial strain for deformation at 293K (**a**) from 0–2.2% and (**b**) from 24–28% total axial compressive strain. The inset in (**a**) exemplifies the increased strain present at the fcc (dendritic) – bcc/B2 (interdendritic) interfaces. The trace of the ${(1\bar{{\rm{1}}}1)}_{{\rm{fcc}}}$ plane is indicated in (**b**).

To study the local strain distribution in fcc vs. bcc/B2 microstuctural components and its evolution with loading at the microscale, samples were incrementally deformed and *ex-situ* high-resolution DIC performed. Figure 5 compares the axial strain (*ε*_*yy*_) maps at the early stage (0–2.2% total compressive axial strain, Fig. 5a) and a late stage (24–28% total compressive axial strain, Fig. 5b) of room temperature deformation. In contrast to single crystals^3,30^, at low total strain distinct macroscopic deformation bands are absent and strain is distributed rather homogeneously across the specimen in both fcc and bcc/B2 phases, as shown by the example in Fig. 5a. However, examination of the DIC strain fields reveals heterogeneous plastic strain accumulations in close proximity to the fcc (dendritic) – bcc/B2 (interdendritic) phase boundaries, as demonstrated by the inset in Fig. 5a. The dendritic-interdendritic interfaces thus act as obstacles for dislocation motion, which can be easily understood as a combined effect of limited coherence across the interface due to different crystal structures and lattice parameters and a misorientation of the associated crystallographic slip (and/or twin) systems.

**Figure 6:**
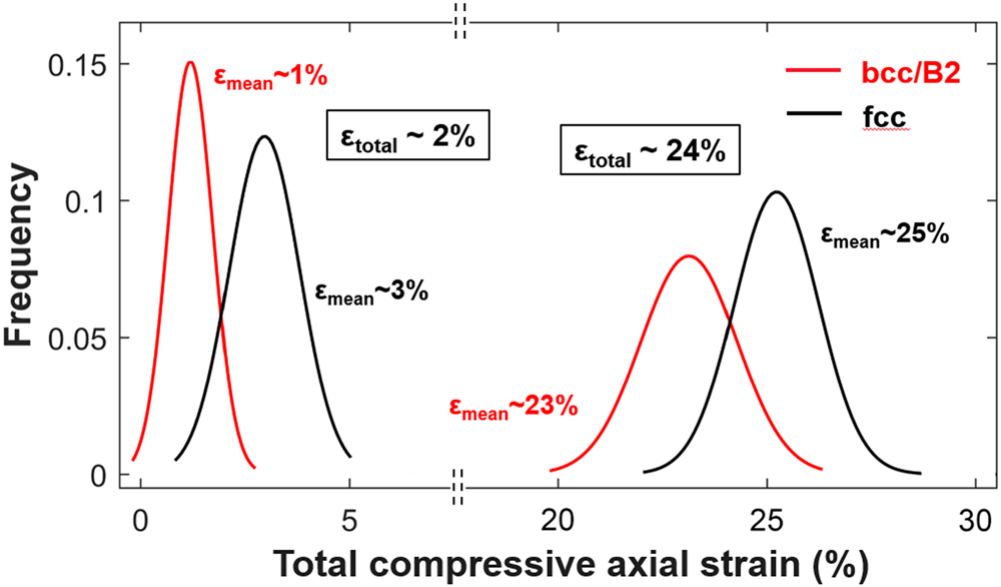
Strain partitioning between fcc and bcc/B2 phases for total compressive axial strains of ~3% and ~24% at 293K.

Figure 6 shows the strain partitioning between fcc and bcc/B2 by plotting the axial strain distributions for both phases as extracted from the DIC maps. The data clearly show that the larger strain fraction is carried by the fcc dendrites. At a total axial strain of ~2% the mean strain in fcc is ~3% while that in bcc/B2 is only ~1%. In other words, the soft fcc deforms more than the hard bcc/B2. Consequently, interdendritic and precipitates prevent the formation of distinct deformation bands and their propagation across the entire sample width as observed in single crystals. These observations share commonality with some recent ones for a closely related Al-rich fcc – bcc dual-phase HEA exhibiting a large bcc volume fraction^26^.

At an advanced stage of deformation (total axial stain ~24%) diffuse deformation bands form inside the fcc and, at least partially, transmit into the interdendritic (Fig. 5b). These bands align with the trace of the ${(1\bar{{\rm{1}}}1)}_{{\rm{fcc}}}$ plane. Still the mean strain in fcc is larger than in bcc/B2 (Fig. 6), albeit the relative differential of the mean strain in fcc and bcc/B2 has shrunk drastically from ~200% at the early stage to ~17% at the advanced stage of deformation. Thus, the bcc/B2 plays an eminent role in carrying the overall plasticity at high strains.

**Figure 7:**
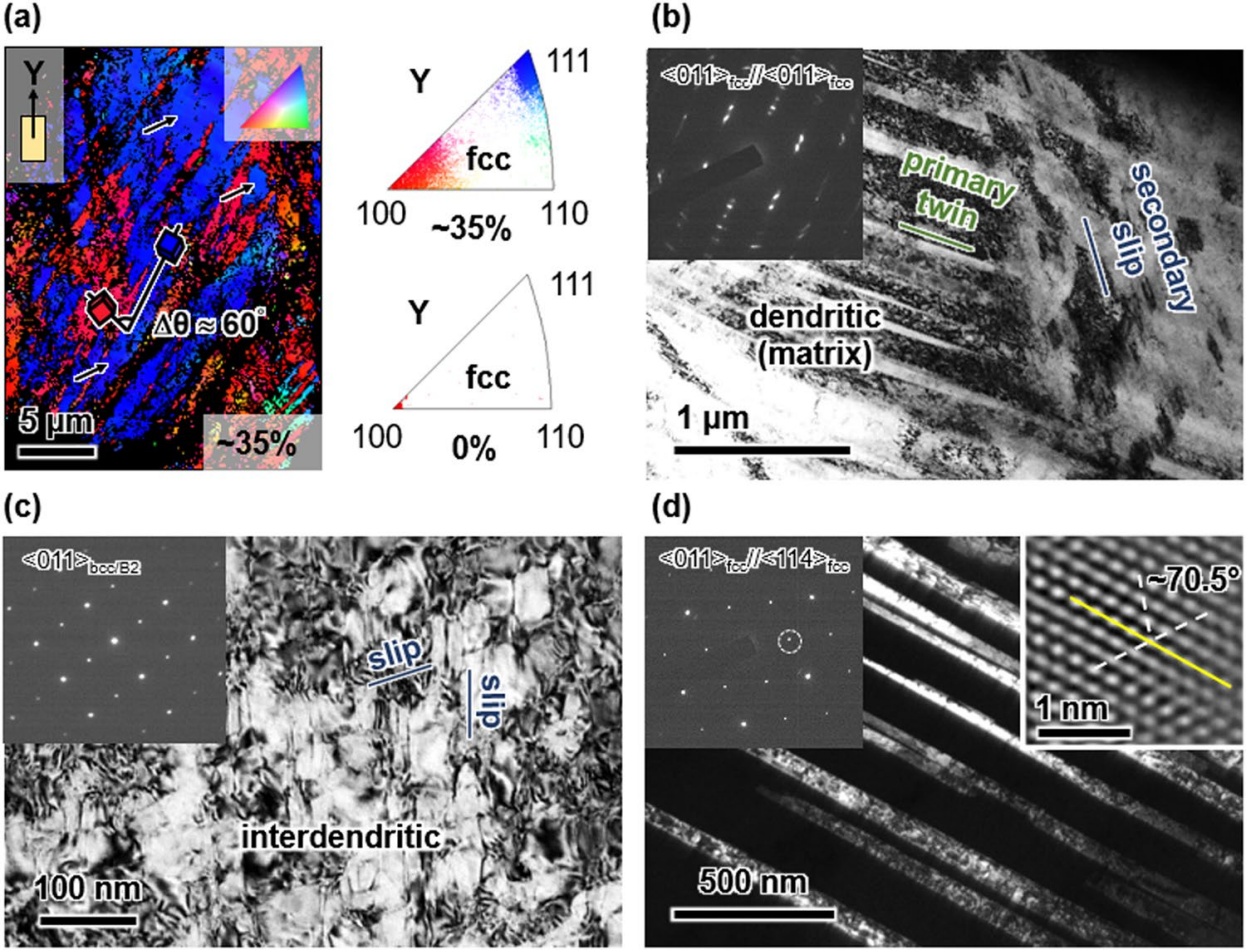
Electron microscopy of 〈001〉_fcc_-oriented FeCoCrNiAl_0.5_ deformed to ~35% total compressive axial strain at 77K and 293K. (**a**) EBSD orientation mapping of the fcc phase deformed at 77K and the corresponding inverse pole figure compared with that for the pristine state. Arrows indicate twins and black pixels represent unindexed areas. (**b**,**c**) Bright-field TEM images of (**b**) slip-twin interaction in the fcc dendrites and (**c**) slip-slip interaction in the bcc/B2 interdendritic after deformation at 77K with diffraction patterns of the corresponding areas. (**d**) Dark-field image of fcc deformation twins after deformation at 293K. The insets show the operative reflection (indicated by a circle in the diffraction pattern) and a high-resolution lattice fringe image of a twin boundary (yellow solid line).

As illustrated in Fig. 4, the macroscopic hardening rate grows substantially by loading beyond 23%, suggesting an additional hardening mechanism, such as twinning, to become active. To test this hypothesis EBSD and TEM were performed on the 77K and 293K deformed FeCoCrNiAl_0.5_ samples after imposing a total axial strain of ~35%. For both testing conditions we discovered fcc deformation twins as illustrated in Fig. 7. At the macroscale, EBSD clearly shows considerable amounts of twinned volumes inside the fcc dendrites (Fig. 7a). In addition, a strong spreading of the sharp pristine 〈001〉_fcc_ orientation due to loading-induced lattice rotation is witnessed. In contrast, below 23% axial strain EBSD did not reveal fcc deformation twins (results not shown here for brevity reasons). TEM confirms the formation of fcc deformation twins (Fig. 7b) above 23% strain and, furthermore, evidences the interaction of secondary systems with primary twins, such as the shearing of primary twins by secondary slip (slip-twin interaction, right half of Fig. 7b). Electron microscopy made further clear that similar to loading at 77K, straining at 293K produces fcc deformation twins likewise (Fig. 7d). The twin nature of the deformation products was verified by high-resolution TEM lattice images in addition to electron diffraction.

The complex stress state inflicted on the dendrite-locked bcc/B2 through external stress and microstructural constraints activates multiple slip systems in it. The TEM-micrograph in Fig. 7c demonstrates this. The slip lines of two non-coplanar system can be seen intersecting, while deformation twins are absent as evidenced by electron diffraction. These slip-slip interactions (forest hardening) further contribute to growing stress levels during straining.

## Discussion

The experimental results presented in the previous section demonstrate the promising yet complex micro-to-macroscale mechanical behavior of dual-phase HEAs on the basis of FeCoCrNiAl_0.5_. DIC and electron microscopy show evidently that strengthening in this class of alloys arises from several factors:

- Dual-phase nature: The fcc + bcc/B2 microstructure consisting of a bcc/B2 interdendritic network and precipitates embedded in fcc dendrites introduces heterointerfaces. Their atomic structure depends on the crystal structures, lattice parameters and the interface orientation relative to the grains separated^35^. The influence of the heterointerfaces is two-fold depending on the bcc/B2 morphology (precipitate vs. interdendritic). While incoming slip can either shear or, due to their small size, circumvent the precipitates (e.g. by Orowan looping or (double) cross-slip) the interdendritic is too extended to be negotiated by these mechanisms. The diffuse nature of deformation bands (Fig. 5b) is in line with the occurrence of cross-slip in the fcc. The interdendritic boundaries impede these deformation bands (slip and/or twin) leading to strain concentrations at the fcc – bcc/B2 interfaces (Fig. 5a). This process, similar to slip transmission across grain boundaries in single-phase polycrystals^36,37^, causes pronounced hardening as it requires additional supply of external stress to transfer plasticity from primary active systems to secondary systems (slip and/or twin).
- Fcc twinning: The fcc dendrites are able to accommodate plastic strain through twinning both at 77K and at room temperature (Fig. 7). Primary twins not only exhibit the outstanding capacity to strongly harden secondary (latent) systems, be they of slip or twin character, but to harden themselves even more. A very recent study^30^ has shown that the slip and twin contributions on the macroscopic hardening rate (d*σ*/dε) differ substantially. It was demonstrated that the self-hardening of twin systems in FeMnCoCrNi single crystals is ~20 times larger than the hardening of latent slip by twin and 50–100 times larger than the self and latent hardening of slip systems among each other. Twin-related interactions are thus by far more important for the overall material strengthening than pure slip interactions. These observations are consistent with the effective stress-strain behavior exhibiting an increased hardening slope above 23% total axial strain (Fig. 4) and the observation of twin-slip interactions in the fcc matrix (Fig. 7).
- Slip activity in bcc/B2: DIC strain maps (Fig. 5) illustrate the accumulation of plastic strain inside the bcc/B2 and, additionally, multiple active slip systems were observed in the bcc/B2 (Fig. 7c). This observation evidences the important role of the bcc/B2 components in carrying the overall deformation^26^. No deformation twinning was observed in the bcc/B2, yet the interaction of primary and secondary slip systems leads to additional hardening. Furthermore, the slip propensity of the bcc/B2 leads to substantial levels of plastic deformability as we like to point out that the samples had not failed before unloading at ~35% total axial strain (Fig. 4).

To differentiate between these contributions to the overall hardening *in-situ* DIC, nanomechanical testing and variation of the amount of heterointerfaces (e.g. via controlling the phase fractions) may provide important insight.

While slip is the most common deformation mode in fcc and bcc materials there are now several high and medium entropy alloys that benefit from deformation twinning and from strain- or pressure-induced martensitic transformation^3,4,10,11,24,38–44^. Especially, the damage tolerance (toughness) profits substantially from these effects. Thus, its improvement is the key driving factor behind the development of novel alloys exhibiting twinning and/or transformation plasticity.

In the present case, extensive twinning leads to a strongly increasing work hardening at high strain (>23% total axial compressive strain) leading to an outstanding hardening rate of ~6 GPa at both room temperature and 77K in the 〈001〉_fcc_-oriented FeCoCrNiAl_0.5_. This behavior contrasts with other dual-phase HEAs, which exhibit high strength and deformability alike, but otherwise steadily decreasing hardening (Fig. 1).

In addition, the dual-phase nature further hardens FeCoCrNiAl_0.5_ generating stress levels exceeding those in single-phase fcc FeCoCrMnNi strained along 〈001〉 by ~50%. Recent studies^30,31^ provided thorough analyses of the loading responses of FeCoCrMnNi single-crystals depending on the load axis orientation. Despite the activation of 3 (non-coplanar) twin systems the stress levels in 〈001〉 FeCoCrMnNi remained below those in 〈001〉_fcc_-oriented FeCoCrNiAl_0.5_, as illustrated in Table 2. Having the same crystallographic orientation the maximal Schmid factors for fcc slip (0.41) and twinning (0.47) are identical in both materials. Twinning was observed on 3 $\{111\}\langle 11\bar{2}\rangle$ systems for compression along 〈001〉 at 77K even from the early stage of deformation on for FeCoCrMnNi. For FeCoCrNiAl_0.5_ only one twin system was found active though, based on TEM (Fig. 7) and DIC (Fig. 5). We therefore surmise that the fine bcc/B2 precipitates play an important role in suppressing twin activity in the fcc-dendrites. Yet, the intricate microstructure of FeCoCrNiAl_0.5_ lifts the stress levels above those of FeCoCrMnNi: 0.93 GPa vs. 0.62 GPa at 5% and 1.1 GPa vs 0.75 GPa at 10% deformation at 77K (Table 2). This is further illustrated by the flow stress of FeCoCrNiAl_0.5_ at 293K being practically identical to the flow stress of FeCoCrMnNi at 77K. Most prominently however, while the maximum hardening rate of both alloy varieties is ~3 GPa below 20% axial strain, it reaches ~6 GPa for FeCoCrNiAl_0.5_ at 35% strain. However, the room temperature hardening slope of FeCoCrMnNi, becomes flat and reduces from 1 GPa at 20% strain to ~0.5 GPa at a higher strain level close to 35%. This particularly steep stress slope in FeCoCrNiAl_0.5_ at high strain can be rationalized by the already high baseline hardening due to the multitude of heterointerfaces and bcc/B2 slip combined with fcc twinning. Wu *et al*. showed that the hardening capacity of twins and their interaction with secondary twin and slip is multiple times higher than of slip alone (up to a factor 100)^30^. The interaction of primary with secondary systems (such as the fcc twin-slip interaction in Fig. 7b or the bcc/B2 slip-slip interaction in Fig. 7c) causes latent hardening of secondary systems. It is well known that a dislocation (lattice or twin) reacting at an interface (twin or hetero) creates a residual dislocation at the interaction site^36,37^. This residual not only forms a barrier for subsequently impinging dislocations but is an indicator for the pertaining strengthening^30,45^. The expected residual magnitudes for dislocations interacting with the misfit dislocation networks at fcc/bcc interfaces can be rather complicated. We thus attribute a central role to dislocation reactions at the fcc - bcc/B2 interfaces and fcc twin boundaries for the superior work hardening of FeCoCrNiAl_0.5_.

**Table 2:** Comparison of key mechanical quantities for dual-phase fcc + bcc FeCoCrNiAl_0.5_ and for single phase fcc FeCoCrNiMn. Flow stress and hardening rates are given for 77K unless specified otherwise.

Phase(s)	Orientation	Composition	Flow stress (GPa)		Max. Schmid factor fcc		Hardening rate (GPa)		Ref.
			5%	10%	slip	twin	20%	35%	
fcc single phase	〈001〉	FeCoCrNiMn	0.62 0.6 (293K)	0.75 0.65 (293K)	0.41	0.47	~3 ~1 (293K)	~0.5 (293K)	^30^
fcc + bcc/B2 dual phase oriented matrix	〈001〉_fcc_	FeCoCrNiAl_0.5_	0.93 0.6 (293K)	1.1 0.75 (293K)	0.41	0.47	~3 (77K and 293K)	~6 (77K and 293K)	this work

Furthermore, work is needed on effects of composition on the intrinsic energy barriers governing slip and twin and latent- and self- hardening due to twin-twin and twin-slip interactions^46^. The present results evidence twinning in FeCoCrNiAl_0.5_ both at 77K and room temperature. In contrast, similar experiments on FeCoCrMnNi single crystals found twinning only at 77K^3,30^. These observations suggest that replacing Mn by Al leads to a beneficial shift of the nucleation stresses for slip and twin, in favor of twinning at room temperature. Building on this, composition optimization may lead the way to develop novel HEAs with even higher work-hardening.

## Conclusions

In the present work, 〈001〉_fcc_-oriented FeCoCrNiAl_0.5_ dual-phase HEA was deformed at 77K and 293K. The deformation mechanisms underlying the stress-strain response were correlated via high-resolution DIC, EBSD and TEM. The most important conclusions drawn from this work are:

1. Strain hardening rates of 6 GPa at high strains (>30%) in dual-phase alloys exceed previously reported hardening levels and open up new possibilities for designing high strength and toughness materials. Central to obtaining superior toughness in dual-phase HEAs is the propensity for deformation twinning of the phases involved. E.g. the particularly high hardening at high strains in the present case can be attributed to twinning of the fcc component.
2. Dual-phase HEA microstructures are conducive to achieving higher strength compared to conventional HEAs due to the multitude of heterointerfaces forming barriers for slip and twinning. Further work is needed to unravel the influence of the minor phase in terms of volume fraction, spatial and size distribution and morphology.
3. Very importantly, the present work illustrates that the full potential of dual-phase HEAs is yet to be explored. The results presented show that in-depth analysis of deformation mechanisms on the micro- and nano-scale in relation to the macroscopic stress-strain and hardening behavior will provide valuable guidance to this end.
